# The Impact of Tetracycline Pollution on the Aquatic Environment and Removal Strategies

**DOI:** 10.3390/antibiotics12030440

**Published:** 2023-02-23

**Authors:** Yernar Amangelsin, Yuliya Semenova, Maryam Dadar, Mohamad Aljofan, Geir Bjørklund

**Affiliations:** 1 Nazarbayev University School of Medicine, Astana 010000, Kazakhstan; 2 CONEM Iran Microbiology Research Group, Tehran 1316943551, Iran; 3 Council for Nutritional and Environmental Medicine (CONEM), 8610 Mo i Rana, Norway

**Keywords:** tetracycline, tetracycline consumption, aquatic environment, tetracycline pollution, oxidative stress

## Abstract

Antibacterial drugs are among the most commonly used medications in the world. Tetracycline is a widely used antibiotic for human and animal therapy due to its broad-spectrum activity, high effectiveness, and reasonable cost. The indications for treatment with tetracycline include pneumonia, bone and joint infections, infectious disorders of the skin, sexually transmitted and gastrointestinal infections. However, tetracycline has become a serious threat to the environment because of its overuse by humans and veterinarians and weak ability to degrade. Tetracycline is capable of accumulating along the food chain, causing toxicity to the microbial community, encouraging the development and spread of antibiotic resistance, creating threats to drinking and irrigation water, and disrupting microbial flora in the human intestine. It is essential to address the negative impact of tetracycline on the environment, as it causes ecological imbalance. Ineffective wastewater systems are among the main reasons for the increased antibiotic concentrations in aquatic sources. It is possible to degrade tetracycline by breaking it down into small molecules with less harmful or nonhazardous effects. A range of methods for physical, chemical, and biological degradation exists. The review will discuss the negative effects of tetracycline consumption on the aquatic environment and describe available removal methods.

## 1. Introduction

Antibacterial drugs are among the most commonly used medications in the world. Antibiotics are antibacterial medications with complex molecular compounds that can destroy or slow the growth of bacteria [1,2]. Antibiotic drugs are classified according to their mechanism of action, spectrum of activity, administration methods, and chemical structure. Antibacterial medications are used for therapeutic purposes and as growth promoters in livestock farming [3,4,5]. It should be noted that the usage of antibiotics is dramatically increasing each year. For example, according to Scaria et al. (2021), the worldwide consumption of antibiotics increased rapidly from 21 to approximately 35 billion daily doses between 2000 and 2015, which is almost a 65% upsurge [6].

Moreover, Klein et al. (2018) predicted that the utilization of antibacterial drugs will continue to grow to 200% by 2030 [7]. The overuse of antibiotics may cause the appearance of antibiotic resistance genes (ARGs) which have negative impacts on human health. ARGs are generated by microbial spontaneous mutations and are selected by antibiotics. This undermines a drug’s antibacterial activity and makes it ineffective in killing bacteria [8,9]. As a result, ARGs can be transferred to other bacteria by horizontal transmission, affecting bacterial communities and developing their resistance to antibacterial medications. The circulation of such strains as methicillin-resistant *Staphylococcus aureus* (MRSA), *Clostridium difficile*, and multidrug resistant *Mycobacterium Tuberculosis* has already caused much harm [10].

One of the most commonly used types of antibiotics currently is tetracycline. The first medications of the tetracycline family were isolated from *Streptomyces* species in the late 1940s. Since then, tetracycline antibiotics have been commercialized owing to their clinical success. The more recent third generation of the tetracycline family demonstrates greater potency and efficacy. Tetracycline inhibits the ability of bacterial protein synthesis by attaching to the 30S ribosomal subunit of bacteria [11,12]. As stated by Fuoco (2012), due to its broad-spectrum activity, this antibiotic can suppress the activity of most Gram-positive and Gram-negative strains, protozoan parasites, including atypical organisms such as mycoplasma, chlamydia, and rickettsia [13]. Due to its low price and robust efficiency, tetracycline is now one of the most commonly used antibiotics.

The overconsumption of antibiotics such as tetracycline in human and animal therapy and livestock has become a major threat to the environment and human health [14]. Tetracycline residue has recently been discovered in a wide range of settings, including soil, surface water, marine environments, sediments, and even biota samples [15]. Tetracycline has negative effects on ecosystems, because it is capable of accumulating along the food chain and causing toxicity to the microbial community, encouraging the development and spread of antibiotic resistance [16]. In addition, tetracycline creates threats to drinking and irrigation water and causes disruption of microbial flora in the human intestine. These detrimental effects raise serious concerns about tetracycline contamination and present an emerging public health issue [17].

Monahan et al. (2022) reported that the weak ability of tetracycline to degrade could cause ecological imbalance [18]. The study highlights the significance of environmental pollution caused by tetracycline, as the outcomes severely affect human health, causing bacterial resistance. It was also stated that an ineffective wastewater treatment system is one of the main causes of antibiotic contamination of the food chain, which might adversely affect human health [19,20,21]. The pollution of soil and water has a drastic negative impact on soil and aquatic microflora [22,23]. This review will assess tetracycline consumption, analyze the antibiotic’s negative impact on the marine environment and aquaculture, and discuss effective methods of tetracycline antibiotic removal from aquatic environments.

## 2. Tetracycline Consumption in Various Fields of Life

Tetracycline is one of the most commonly used types of antibiotics in the world. It has broad-spectrum activity against various bacterial infections, making it effective in human therapy and veterinary medicine [24]. The indications for treatment with tetracycline include infectious diseases such as pneumonia, bone and joint infections, infectious disorders of the skin and sexually transmitted infections, as well as gastrointestinal infections. Tetracycline is a potent agent against the so-called biothreat pathogens, such as *Bacillus anthracis*, *Yersinia pestis*, and *Francisella tularensis*, which are the causative pathogens of some of the most lethal infections. In general, tetracycline-class drugs are the first-line treatment options for many infectious diseases [25].

It was estimated that humans’ daily consumption of tetracycline worldwide is 23 kg [26]. It was also reported that European countries and Switzerland use almost 2300 tons of tetracycline antibiotics for animal farming, approximately 66% of the total number of antibiotics [27]. According to the statistics provided by Xu et al. (2021), tetracycline antibiotics are the third most often used drugs in Brazil after penicillin and quinolones [28]. It is estimated that more than 2500 tons of tetracycline are consumed yearly for animal therapy in Europe [29].

Moreover, agriculture and aquaculture are some of the main fields of tetracycline consumption, as antibiotics are used for animal growth. According to Ahmad et al. (2021), 180 thousand tons of raw antibiotic materials were used in China for human treatment and farming, which is equivalent to 138 g per capita per year [30]. It was stated that 172 mg, 148 mg, and 45 mg of antibiotics per kilogram were administered to slaughtered pigs, chickens, and cows [31]. By the calculations of the study provided in pig and cattle livestock in China, it was stated that the daily antibiotic excretion of a single pig and cow is 18.2 and 4.24 mg, respectively [32]. Wozniak-Biel et al. (2018) state that poultry and livestock animal farming used 33% of total tetracycline antibiotics in Turkey [33].

Meanwhile, the USA manufactures 22,680 tons of antibiotic formulations annually, 40% of which is used for agriculture purposes [34]. As a result, tetracycline contamination has become an emerging issue for the environment and human use. Furthermore, the study by Mahamallik et al. (2015) provided another source for tetracycline contamination from the waste of pharmaceutical industries [35]. This study indicates that unutilized antibiotics are distributed to unsuitable places for waste products with no treatment procedures. Therefore, antibiotics will remain undegraded for years in soil or water sources.

**Figure 1:**
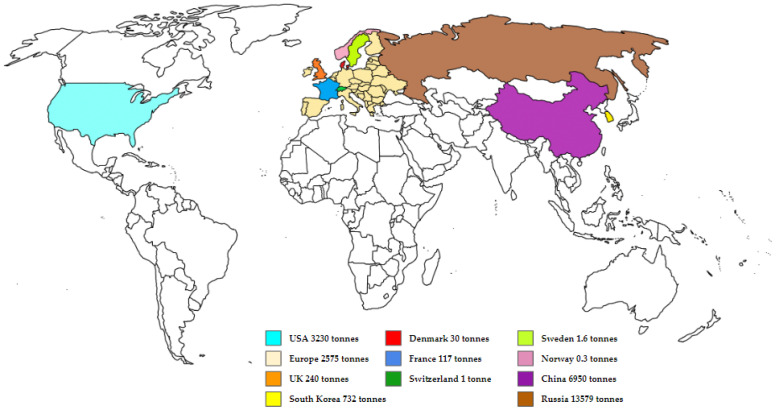
Tons of tetracycline consumed in different countries.

Figure 1 represents the available data on consumption of tetracyclines in different parts of the world. Tetracyclines are widely used in European Union countries (2575 tons consumed annually) and Switzerland (1 ton consumed annually) [36]. Three other countries with high levels of tetracycline consumption are Russia (13,579 tons per year) [37] China (6950 tons per year) [38] and the USA (3230 tons) [39]. According to Xu et al., China alone accounts for 18% of global consumption [28]. South Korea consumes 732 tons of tetracyclines, and the UK consumes 240 tons every year [39]. These considerably large proportions of tetracyclines could be explained by the ampleness of animal livestock; therefore, tetracycline is used as a feed promoter. The negative consequences associated with excess tetracycline consumption are discussed in the following paragraphs.

## 3. The Adverse Effect of Tetracycline Antibiotics on the Aquatic Environment

The most common reason for tetracycline pollution is its stability and low metabolism in human and animal organisms. As stated by Xu et al. (2021), tetracycline antibiotics cannot be fully absorbed and metabolized in the body [28]. Therefore, approximately 75% of the antibiotic is excreted in its parenteral form [40,41]. Tetracycline contamination has been identified primarily in water sources, which causes environmental pollution in the surrounding area, damaging the ecological system [42,43,44]. Overconsumption of this antibiotic in human and animal therapy and its utilization in agriculture as a growth promoter are among the main causes of tetracycline pollution in the aquatic environment [28].

Moreover, tetracycline is also used in aquaculture for fish feeding. It can be assumed that approximately 80% of utilized antibiotics in aquaculture will be freed in the aquatic environment [45]. It has been estimated that the concentration of tetracycline antibiotics is high in rivers due to pharmaceutical manufacturing, its usage in hospitals, and facilities for animal management [46,47]. This situation harms aquaculture in the face of bacterial resistance due to misuse or overuse of tetracycline in fish farming [48,49]. As a result, bacteria in water and fish pathogens might develop antibacterial resistance.

### 3.1. The Adverse Effects of Tetracycline on Algal and Plankton Communities

Tetracycline is harmful for algal communities, inhibiting growth of different algae in the concentration range 0.25–30 mg/L. It is not surprising that the higher the dose, the more profound are the effects. Such, 94% growth inhibition of mixed algae is observed at a concentration of 30 mg/L. Eukaryotic algae species are more vulnerable to tetracycline [50]. Freshwater green algae are sensitive both to tetracycline and its degradation products. According to Xu et al., exposure to tetracycline and its metabolites increases the permeability of algal cells and causes structural changes, including plasmolysis, starch granule deposition, deformation of the thylakoid lamellae in the chloroplasts, and enlargement of the vacuoles. These effects are more profound at higher tetracycline concentrations (>5 mg/L) [51]. The ability of tetracycline to impact the protein synthesis machinery stands behind these alterations [52].

Tetracycline also affects phytoplankton and zooplankton communities in a dose-dependent manner. These effects include reduction in abundance and species richness, which recover after exposure to tetracycline is discontinued. In addition, elevated concentrations of tetracycline decrease water clarity and lower levels of dissolved oxygen [53]. Tetracycline is capable of inducing the cyanobacterial bloom increasing the density of bacteria more than two-fold. The shift observed is in favor of *Synechococcus, Microcystis*, and *Oscillatoria* and against eukaryotic microalgae [54].

### 3.2. The Adverse Effects of Tetracycline on Fish Community

Apart from algae and plankton, tetracycline has many negative effects for the fish community, among which is embryotoxicity. Zebrafish (Danio rerio) have recently gained popularity as a potential animal model for research into the toxicity of various antibiotics, including tetracycline. Zhang et al. (2015) identified the toxic impact of tetracycline antibiotics on zebrafish embryos. It was stated that the drug could activate cell apoptosis by causing oxidative stress, which impedes the development of zebrafish embryos. Additionally, tetracycline induces caspase-dependent apoptosis in the early stages of zebrafish [55].

Oliveira et al. (2013) assessed the effects of tetracycline on zebrafish development and concluded that it causes delayed hatching of embryos [56]. According to Yu et al. (2020), prolonged exposure of zebrafish embryos to tetracycline at environmentally relevant concentrations causes elevated transcription of genes involved in thyroid synthesis, which might lead to thyroid dysfunction. Moreover, the authors reported that with an increase in exposure to tetracycline, zebrafish showed a decline in body length, weight, and BMI [57]. The sensitivity to tetracycline appears to vary depending on the developmental stage. Zebrafish embryos absorb more tetracycline at 3 days postfertilization than at 6 h postfertilization. Exposure to tetracycline at 0.4 mg/mL results in zebrafish death [58].

Jia et al. (2020) investigated the impact of tetracycline on aquatic culture. They conducted a study on the goldfish *Carassius auratus* (Linnaeus, 1758) treating them with tetracycline antibiotics at concentrations that are real to environmental conditions. This study discovered the negative impact of tetracycline on fish gut microbial flora. Jia identified the adverse effects of treatment with tetracycline on different *Aeromonas* species tolerance and changes in bacterial communications due to exposure to tetracycline. According to their results, tetracycline stress dramatically increased the resistance ratio in cultivated gut bacteria, and there was growth in antibiotic tolerance of *Aeromonas* species [59]. Coincidentally, there was a significant change in the structure of the gut microflora of goldfish and the abundance of antibacterial resistance genes, which can encode efflux of tetracycline antibiotics as a result of treatment with tetracycline [59].

Another dangerous effect of tetracycline on aquatic culture was found in the study of Yu et al. (2019). A study showed that tetracycline antibiotics cause antioxidative stress in fish organisms [60]. The stress response of an organism is protection caused by external stimuli in the face of variations in hormone levels, energy metabolism, motor control, and electrolyte balance [61]. For instance, in fish organisms, cortisol is one of the main significant stress indicators that can regulate the response to stress and glucose metabolism [62].

Yu et al. (2019) discovered that glucose, an energy source and intermediate of metabolism, was drastically decreased in zebrafish due to exposure to tetracycline, while significant alterations were not shown in cortisol levels. Consequently, it is assumed that fish require more energy sources to maintain the excessive level of reactive oxygen species (ROS) production by tetracycline antibiotics [60]. Treatment with tetracycline dramatically decreases glucose and NrF2 mRNA and protein levels in zebrafish larvae. Moreover, tetracycline molecules could dock with more stable hydrogen bonds into the binding pocket of PI3K of zebrafish larvae, an important protein that activates NrF2. In conclusion, tetracycline antibiotics could notably induce oxidative stress responses in zebrafish larvae. Additionally, tetracycline inhibits the activation of NrF2 and reduces the capacity of antioxidation by inhibiting the PI3K enzyme [60].

Another long-term effect of exposure to tetracycline is alterations in fish behavior. As Almeida et al. (2019) identified that exposure of zebrafish to low concentrations of tetracycline results in increased exploratory behavior. Interestingly, tetracycline treatment induced photosensibility that changed the swimming pattern of zebrafish. These effects were partially reversible after the exposure was discontinued [63]. Petersen et al. (2021) also reported alterations in zebrafish behavior following acute exposure to tetracycline. These changes included impaired locomotor activity, memory/learning processes, and proneness to aggressive behavior [64]. Several mechanisms could be proposed to explain alterations in fish behavior following tetracycline exposure. From one side, tetracycline leads to intestinal dysbiosis and this might influence the gut brain axis of fish. From the other side, prolonged exposure to tetracycline alters the levels of triiodothyronine and thyroid-stimulating hormone which might be the sign of the hypothalamus–pituitary–thyroid axis involvement. In addition, a decrease in cortisol levels is observed after tetracycline exposure, which impacts anxiety-related behaviors [65].

Concerning the above, it could be concluded that tetracycline pollution harms the aquatic environment and culture, affecting fish embryonic development and the gut microbiome, altering fish behavior, and causing oxidative stress. Tetracycline pollution negatively affects other aquatic organisms, apart from fish. Acute and chronic exposure to tetracycline may lead to an increased prevalence of diseases related to the digestive, nervous and immune systems of fish, which might also be influenced by the development of antibiotic resistance. 

### 3.3. Development of Antibiotic Resistance in Bacteria

It was estimated that 90% of bacteria in the aquatic environment are resistant to at least one antibiotic and 20% are multi drug resistant [66]. Bacteria develop antibiotic resistance via molecular and genetic pathways. There are four well-defined mechanisms: efflux pumps, tetracycline-inactivating enzymes, ribosomal protection proteins and spontaneous mutations in target genes. Approximately 50 tetracycline-resistance genes are known up to now, which are typically encoded in plasmids and transposons (some tetracycline genes reside on chromosomes) and are passed from one species to another [67]. Once provoked, antibiotic resistance spreads rapidly among aquatic microbial populations eventually reaching human pathogens by means of horizontal gene transfer. This results in the appearance of resistant strains that are more difficult to treat with available antibiotics and increase in the occurrence of infectious diseases, which also tend to be more severe [68]. 

More research is needed to demonstrate a link between molecular events and physiological as well as pathological effects of tetracycline exposure in fish and other aquatic organisms. In addition, it is important to investigate the ability of aquatic organisms to recover from tetracycline exposure. Since the contamination of aquatic environments with antibiotics is likely to grow, there is a need to search for methods that can effectively remove them.

## 4. Effective Methods of Tetracycline Removal from the Aquatic Environment

As discussed above, tetracycline pollution has seriously threatened human health and the environment. Ineffective wastewater systems are one of the main reasons for the increased antibiotic concentrations in aquatic sources [69]. Moreover, Ahmad et al. (2021) state that oxidation of tetracycline is difficult in the environment due to its stable compound. Tetracycline can also be unstable at low pH because of their epi- and anhydrous-product formation, but it makes them less degradable due to their low volatility [30]. Another reason for the difficulty of tetracycline removal from water sources is because of its ability to form stable compounds by binding Ca^2+^ and other ions [70,71]. However, some methods can degrade tetracycline by breaking it down into small molecules with less harmful or nonhazardous effects.

A number of approaches have been developed for the degradation of pharmaceuticals from the aquatic environment. Thus, there is a range of traditional treatment methods used for removal purposes. These include biological, chemical and physical removal, such as membrane filtration, coagulation, prechlorination, adsorption, and flocculation [72]. These methods have many benefits but also drawbacks, including maintenance requirements and cost considerations [73]. 

### 4.1. Removal of Tetracycline by Adsorption

In general, adsorption of tetracycline is a relatively inexpensive and simple method. Adsorption techniques are increasingly used to remove organic particles from contaminated streams. Currently, various adsorbents are available, such as chitosan particles, graphene oxide, kaolinite, magnesium oxide, smectite clay, rectorite, aluminum oxide, palygorskite, coal humic acid, activated carbon, and others [74]. Accumulation of a contaminant from liquids/gases to the adsorbent surface is the nature of adsorption process. The efficiency of adsorption depends on the adsorbent properties, i.e., porosity (both at micro and macro levels), pore diameter, and specific surface area. For chemical adsorption, functional groups also play an important role. Several adsorption mechanisms participate in removal of antibiotics from liquid media: electrostatic attraction, pore-filling, partition into uncarbonized fractions, hydrophobic interaction, surface precipitation and formation of hydrogen bonds [75].

Yu et al. utilized oxidized multi-walled carbon nanotubes to remove tetracycline from water and found out that chemical properties of aqueous solution (ionic strength, pH, and the presence of Cu^2+^) play an important role in tetracycline adsorption [76]. As the application of adsorbents for tetracycline removal generates waste, photodegradation or other techniques are commonly used for pollutants removal from adsorbents. Bhangi and Ray applied kappa-carrageenan and iron oxide nanoparticle-filled poly composite gel with subsequent photocatalytic degradation to remove tetracycline from water. The solution pH, dose of the polymer, and initial concentration of tetracycline strongly influenced the efficiency of tetracycline removal. High regeneration and recyclability were among the benefits of nanocomposite adsorbent [77]. 

Biochars are carbon-based adsorbents that have many advantages, such as low cost, large surface area and ease of regeneration. Natural biomasses, including solid wastes, could be used for their production. Biochars appear to be ideal adsorbents, owing to enriched surface functional groups. In most instances, biochars do not require additional activation processing, although it could be performed [75]. The interest in the application of biochars for tetracycline removal increased over the past decade reflecting intense research efforts. Seaweed biochar derived from *Sargassum species* was utilized by Song et al. to adsorb tetracycline from water. The uptake of tetracycline decreased with increasing pH and the removal efficiency of biochar regenerated in different solutions could be as high as 91.2% [78]. Shrimp shell waste can also be used for biochar production, as reported by Chang et al. This environmentally friendly adsorbent removes high concentrations of tetracycline very efficiently and the maximum adsorption capacity was 229.98 mg/g for 36 h at 55 °C [79]. Chitosan and its composites appear to be another attractive biochar that can be produced from seafood wastes by chitin extraction. It can be used alone or in combination with other adsorbents, such as carbon nanotubes and graphene oxide. Da Silva Bruckmann et al. investigated the utility of magnetic chitosan (CS·Fe_3_O_4_) to remove tetracycline after several adsorption/desorption cycles. The authors concluded that adsorption capacity is influenced by several factors, such as initial concentration of tetracycline, adsorbent dosage, ionic strength, and pH [80]. However, translational studies are needed to evaluate the performance of adsorption methods in practical settings.

### 4.2. Removal of Tetracycline by Photodegradation

One of the methods of tetracycline removal is an advanced oxidative process (AOP). This process requires the reaction between hydroxyl radicals (•HO), which have high oxidative reactivity, and organic compounds [81]. These highly reactive hydroxyl radicals can be generated by hydrogen peroxide, ozone, and metal or semimetal catalysts. AOP can produce substrates with low toxicity and then mineralize them [82]. Advanced oxidation processes can be divided into techniques such as Fenton, ozonation, and UV photolysis, depending on the catalysts used and ultrasounds [83]. One of the effective oxidizing agents for tetracycline degradation is ozone. Ozone can react with tetracycline in protonated form by adding one oxygen atom at C11a-C12 and two oxygen atoms at C2-C3 [84]. Even though ozonation is considered to be effective for tetracycline removal, several factors, such as pH and TC concentration, might impact its efficacy. The study by Ahmad et al. (2021) suggests that the pH level has a crucial influence on the ozonation process. If the pH is low, then the hydroxyl group at ring I of tetracycline cannot be dissociated, which decreases electron densities in the C2-C3 locations. As a result of reduced electron densities, the possibility of ozone reacting with the C2-C3 position will decline [30,85]. Therefore, it will negatively affect the effectiveness of the degradation process. 

Although tetracycline does not degrade under visible light, it can be degraded via exposure to ultraviolet irradiation at both 254 and 185 nm wavelengths. This approach enables disinfection and removal of other pollutants, apart from tetracycline. In general, exposure to 185 nm UV resulted in better degradation of tetracycline than exposure to 254 nm UV. This could be explained by the ability of 185 nm UV to cause the photodissociation of water molecules and direct photolysis [86]. As a result, highly reactive hydroxyl radicals are formed in elevated concentrations. Moreover, the presence of dissolved oxygen increases degradation rates by an additional 16%, which is probably associated with the increased formation of oxidative radical species. The combination of UV with hydrogen peroxide or Fe^2+^ also improves the degradation of tetracycline, as well as the combination with ozone and hydrogen peroxide [87]. Fortification of UV radiation by application of peroxomonosulfate or persulfate helps to increase the rate of tetracycline degradation. This is also associated with the generation of oxidative radical species (both hydroxyl radicals and SO_4_^2−^). The resulting removal efficiency may even exceed 80% [88]. Table 1 presents the effectiveness of adsorption and photodegradation methods in the removal of tetracycline from aquatic environments.

### 4.3. Removal of Tetracycline by Physico-Chemical Methods

Once physical and chemical methods proved to be successful in the removal of tetracycline from water and wastewater, it is reasonable to conclude that a combination of these methods will enable higher clearance. Sonolysis is a physico-chemical process and is an efficient method for the full mineralization of wastewater. This is a relatively new advanced oxidation process. Ultrasound and associated cavitation produce both physical and chemical effects. The chemical effects of ultrasound involve the generation of high temperature and pressure, which result in the formation of various oxidizing species, such as hydrogen, hydroxide, hydroperoxide, hydrogen peroxide, etc. These are very active oxidizing species that can instantly attack the organic molecules in the majority of refractory organic contaminants and destroy them. The physical effects of ultrasound mostly include intense mixing or convection in the medium, resulting in microstreaming [89].

Hou et al. (2016) reported their experience utilizing the coupled ultrasound/Fenton-like process to degrade tetracycline over an Fe_3_O_4_ catalyst. According to the study findings, the application of ultrasound considerably increased the catalyst’s stability. After 60 min of treatment, 93.6% of tetracycline was eliminated under ideal circumstances [90]. Wang et al. (2011) used ultrasound-amplified catalytic ozonation by a goethite catalyst in an air-lift reactor. The authors reported a 100% removal of tetracycline, which was influenced by the high concentration of gaseous ozone (13.8 mg/L), increasing gas flow rate and power density (ultrasound frequency was 20 kHz and power was 250 W) [91].

Over the past decade, photocatalytic strategies for tetracycline removal have been extensively studied in combination with ultrasound. P25 titanium dioxide nanopowder was used to induce photocatalytic degradation with simultaneous application of hydrodynamic cavitation. The highest degradation of tetracycline was 78.2%, and the reaction time was 90 min [92]. Ghoreishian et al. (2019) reported sonophotocatalytic degradation of tetracycline with the help of reduced graphene oxide/cadmium tungstate composite hierarchical structures. The authors stated a complete degradation of tetracycline (the initial concentration was 13.54 mg/L). The overall time of ultrasound-assisted reaction equaled 1 h, and the catalyst loading was 0.216 g/L [93]. Heidari et al. (2018) used a composite photocatalytic material (Bi_2_Sn_2_O_7_-C_3_N_4_/Y) for the degradation of tetracycline. The maximum rate of tetracycline degradation was 80.4%, and the concentration of Bi_2_Sn_2_O_7_-C_3_N_4_/Y zeolite was 1 g/L with a reaction time equal to 90 min [94].

Although the above-described methods of physico-chemical removal of tetracycline have many advantages, primarily high effectiveness, they may also yield dangerous transformation byproducts. The photolysis technique is capable of producing even more toxic intermediates than tetracycline itself [95]. In addition, some of them pose high requirements for reagents or have increased energy demands. Therefore, the development of biological methods for tetracycline removal appears to be a promising approach. Table 2 summarizes the results of different studies on the effectiveness of physico-chemical methods used for tetracycline removal from aquatic environment.

### 4.4. Removal of Tetracycline by Biological Methods

Biodegradation and biosorption are two main biological methods used to remove antibiotics from the aquatic environment. Microbial metabolism and cometabolism are both a part of biodegradation. In microbial metabolism, microorganisms use antibiotics as carbon sources and energy substrates for their growth. Meanwhile, in the case of microbial cometabolism, antibiotics can be destroyed by homologous enzymes released by the microbial population. Biosorption applies to the removal of antibiotics by electrostatic and hydrophobic interactions [96].

Enzymes are frequently used for the treatment of various contaminants. They are characterized by mild reaction conditions, rapid reaction times, high efficiency, and minimal energy consumption [97]. It was demonstrated that horseradish peroxidase can remove 50% of tetracycline within 1 h [98]. Meanwhile, *Phanerochaete chrysosporium* expresses three key ligninolytic enzymes: lignin peroxidase, manganese peroxidase, and laccase. Sun et al. (2021) utilized manganese peroxidase from *Phanerochaete chrysosporium* to biodegrade tetracycline and showed that it can transform as much as 80% of tetracycline within 3 h [99]. Yang et al. (2017) developed a review on the potency of laccases to remove tetracycline [100]. According to Becker et al. (2016), the use of immobilized laccase from *Trametes versicolor* helped to remove >70% of tetracycline within 24 h [101]. Wen and Li (2009) used crude lignin peroxidase from *Phanerochaete chrysosporium* to biodegrade tetracycline. This enzyme can remove up to 95% of tetracycline within 5 min under optimal conditions [102].

There is a series of computational studies that model the ability of different enzymes to biodegrade antibiotics, including tetracycline. Currently, bioinformatics presents an economically viable way to predict the molecule’s properties prior to experimental trials. For this purpose, molecular docking and molecular dynamics analyses are applied. Cárdenas-Moreno et al. (2019) modeled the interactions between laccases from *Ganoderma weberianum* and tetracycline. The root mean square deviation of the laccase-tetracycline interaction was 1.991. As lower values indicate higher similarity, it may be concluded that laccase can effectively bind to tetracycline [103].

Several mechanisms could be proposed to explain how microorganisms can remove tetracycline via biodegradation. Microorganisms can biodegrade tetracycline by opening loop structures and cutting functional groups, such as N-methyl, carbonyl, and amino groups [104,105,106]. Tetracycline is degraded by a range of microorganisms, including *Bacillus* sp., *Stenotrophomonas maltophilia, Klebsiella* sp., *Sphingobacterium* sp., *Trichosporon mycotoxinivorans, and Shewanella species* [107]. *Stenotrophomonas maltophilia* has been linked to a number of potential degradative pathways, and processes such as decarbonylation, denitromethylation, and deamination have all been identified [108]. Similarly, *Klebsiella* sp. uses hydrolysis ring-opening, oxidation, deamination, decarbonylation, and demethylation reactions in the process of tetracycline degradation [109]. At the same time, *Trichosporon mycotoxinivorans* utilizes proton-transfer pathway reactions, dehydration, and epimerization as parts of the tetracycline breakdown process [30].

A novel method of wastewater treatment known as a membrane bioreactor (MBR) combines the conventional activated sludge process with membrane separation technology. According to Tran et al. (2016), MBRs can be implemented for treating wastewater for the removal of tetracycline. It was demonstrated that acclimated sludge from an MBR can degrade up to 40 mg tetracycline with 83.3–95.5% antibiotic removal compared to the conventional process with 44.3–87.6% efficiency [106]. Xu et al. (2019) showed that MBRs can remove more than 90% of tetracycline via both biodegradation and adsorption [110]. Sheng et al. (2018) proved that MBRs have high removal efficiency for tetracycline (87.6–100%) at environmentally relevant concentrations (1 mg/L). Nevertheless, it had inadequate removal at higher tetracycline concentrations (i.e., 10 mg/L) [111]. These data illustrate that tetracycline removal by membrane bioreactors is an effective option for wastewater treatment, but only if the concentration of tetracycline is relatively low. 

On the basis of all the above stated, it might be concluded that there is a need for the continuous search and development of innovative methods of tetracycline removal from various aquatic environments. This will help to overcome the issue of environmental pollution caused by tetracycline and minimize the associated negative effects. The treatment of wastewater requires special attention. Table 3 summarizes the effectiveness of biological methods used for tetracycline removal.

## 5. Conclusions

Tetracycline is one of the most common antibiotics used for human and animal therapy in agriculture and aquaculture to promote growth. Globally, aquatic environments are polluted by tetracycline due to its widespread use and high stability. This promotes antibiotic resistance and necessitates development of novice antibacterial drugs. In addition, tetracycline has many toxic effects on aquatic organisms and breaks the equilibrium, causing dysbiosis. The negative effects of tetracycline are dose-dependent and there is a need for continuous monitoring of tetracycline pollution in different aquatic systems. A range of methods for tetracycline removal has been proposed and future research needs to focus on evaluation of their effectiveness in real world practice. Antibiotic pollution of wastewaters is another major concern. Biodegradation appears to be a promising method to solve this issue, yet a lot needs to be done before it becomes a routine approach in conventional wastewater treatment plants.

## Figures and Tables

**Table 1:** Studies on the effectiveness of adsorption and photodegradation methods in removal of tetracycline.

First Author, Year of Publication, Reference	Initial Concentration of Tetracycline	Matrix	Removal Method	Reported Effectiveness
Yu et al., 2014 [76]	7.3–151.6 mg/L^−1^	Deionized water	Adsorption (Oxidized multi-walled carbon nanotubes)	The removal rate approximated 70% (at pH range of 3.3–8.0).
Bhangi and Ray, 2022 [77]	50–300 mg/L^−1^	Deionized water	Adsorption and photocatalytic degradation by kappa-carrageenan and iron oxide nanoparticle-filled poly composite gel	The efficiency of photo-degradation was 86.1% after 2 hours.
Song et al., 2019 [78]	500 mg/L^−1^	Deionized water	Adsorption (biochar derived from seaweed)	The removal efficiency ranged from 89.2 to 91.2%.
Chang et al., 2020 [79]	400 mg/L^−1^	Deionized water	Adsorption (biochar derived from shrimp shell waste)	The maximum adsorption capacity was 229.98 mg/g for 36 h at 55 °C.
Da Silva Bruckmann et al., 2022 [80]	10–200 mg/L^−1^	Deionized water	Adsorption (magnetic chitosan, CS·Fe_3_O_4_)	The highest adsorption capacity reached 211.21 mg/g^−1^ (at pH 7.0).
Dalmázio et al., 2007 [84]	52.8 mg/L^−1^	Deionized water	Ozone/air gas stream	Almost complete degradation after 120 min.
Gulnaz et al., 2016 [85]	400 mg/L^−1^	Deionized water	Ozone/air gas stream	Complete removal after 40 min.
Luu and Lee, 2014 [87]	20 mg/L^−1^	Artificial wastewater	Ozone/ultraviolet, Ozone/hydrogen peroxide/ultraviolet	Complete removal.
Xu et al., 2020 [88]	18.22 g/L^−1^	Natural water (tap water, Xincheng river and Taihu lake)	Ultraviolet C, Ultraviolet C/persulfate	The removal efficiency exceeded 80%.

**Table 2:** Studies on the effectiveness of physico-chemical methods in removal of tetracycline.

First Author, Year of Publication, Reference	Initial Concentration of Tetracycline	Matrix	Removal Method	Reported Effectiveness
Hou et al., 2016 [90]	100 mg/L^−1^	Deionized water	Ultrasound/heterogeneous Fenton process	93.6% of tetracycline was removed after 60 min.
Wang et al., 2011 [91]	100 mg/L^−1^	Deionized water	Ultrasound/goethite/ozone	Complete removal.
Wang et al., 2017 [92]	30 mg/L^−1^	Deionized water	Photocatalysis/hydrodynamic cavitation	78.2% removal after 90 min.
Ghoreishian et al., 2019 [93]	13.54 mg/L^−1^	Deionized water	Sonophotocatalysis	Complete removal (after 60 min).
Heidari et al., 2018 [94]	10–30 mg/L^−1^	Deionized water	Sonophotocatalysis	80.4% degradation after 90 min.

**Table 3:** Studies on the effectiveness of biological methods in removal of tetracycline.

First Author, Year of Publication, Reference	Initial Concentration of Tetracycline	Matrix	Removal Method	Reported Effectiveness
Leng et al., 2020 [98]	0.13 mg/L^−1^	Wastewater collected from the Tudhoe Mill Sewage Treatment Plant, UK	Enzymatic treatment with horseradish peroxidase, horseradish peroxidase/redox mediator	The mean degradation was 47.57% after 30 min and 67.90% after 8 h.
Sun et al., 2021 [99]	10–50, 100 mg/L^−1^	Pure water	Enzymatic treatment with manganese peroxidase	The degradation rate was 80% (<50 mg L^−1^) and 60% (≥50 mg L^−1^).
Becker et al., 2016 [101]	10 mg/L^−1^	Deionized water	Enzymatic treatment with fungal laccase	70% removal within 24 h.
Wen et al., 2009 [102]	50 mg/L^−1^	High purity water	Enzymatic treatment with lignin peroxidase	95% removal after 5 min.
Tran et al., 2016 [106]	Median concentration 3604 ng/L^−1^	Wastewater from a conventional wastewater treatment plant	Conventional activated sludge, membrane bioreactor	Membrane bioreactor removed 83.3–95.5% and conventional process had 44.3–87.6% efficiency.
Xu et al., 2019 [110]	1000 μg/L^−1^	Wastewater from a conventional wastewater treatment plant	Membrane bioreactor	90% of tetracycline was removed.
Sheng et al., 2018 [111]	1, 10, 100 μg/L^−1^; 1, 10 mg/L^−1^	Activated sludge from a conventional wastewater treatment plant	Nitritation membrane bioreactor	The removal rate was 87.6–100% at low concentration (≤1 mg/L) but poor at higher concentration (≥10 mg/L).

## Data Availability

Not applicable.
